# Is cooperation better than competition? Evidence from patient mobility before and after the COVID-19 pandemic in Tuscany, Italy

**DOI:** 10.3389/fpubh.2025.1551574

**Published:** 2025-07-30

**Authors:** Giovanni Guarducci, Davide Golinelli, Luca Spadea, Giuliana Fabbri, Nicola Nante

**Affiliations:** ^1^Post Graduate School of Public Health, University of Siena, Siena, Italy; ^2^Department of Life Sciences, Health and Healthcare Professions, Link Campus University, Rome, Italy; ^3^Healthcare Management, University Hospital Trust of Ferrara, Ferrara, Italy; ^4^Department of Molecular and Developmental Medicine, University of Siena, Siena, Italy

**Keywords:** hospital patient mobility, health policy, Italian National Health Service, regional strategies, cross-border mobility, DRGs

## Abstract

**Introduction:**

According to Italian Law, citizens have the right to choose the preferred place of care; in this context, the competition between various hospitals across the national territory is inevitable. The study aims to analyze the associations between patient mobility between Tuscany (Italy) and other Italian regions and the weight of Diagnosis Related Groups (DRGs) provided for these hospitalizations, as a proxy of complexity of care provided.

**Methods:**

A retrospective study was conducted using 2019 and 2022 Hospital Discharge Cards data of Tuscany provided by the Italian Ministry of Health. The surgical DRGs of each Major Diagnostic Categories (MDCs) were considered according to high specialty (HS) and non-high specialty (N-HS). For each MDCs, patient attractions and escapes to bordering and non-bordering regions with Tuscany were associated with HS and N-HS surgical discharge DRGs by using Odds Ratios (OR). The analysis was performed thought STATA software, and the significance level was set at 95%.

**Results:**

In studied period, for Digestive Diseases (in 2019 OR: 1.37; 95%CI: 1.08–1.74; in 2022 OR: 1.66; 95%CI: 1.29–2.12), patients from non-border regions were more likely to be attracted to the Tuscany hospitals for HS DRGs, while for Musculoskeletal Diseases (in 2019 OR: 0.51; 95%CI: 0.47–0.56; in 2022 OR: 0.53; 95%CI: 0.48–0.59), those from non-border regions were less likely to be attracted for HS DRGs (*p* < 0.05). For the same years, for Nervous Diseases (in 2019 OR: 3.08; 95%CI: 2.18–4.37; in 2022 OR: 2.27; 95%CI: 1.60–3.22) and for Hepatobiliary and Pancreas Diseases (in 2019 OR: 4.56; 95%CI: 2.71–7.68; in 2022 OR: 4.19; 95%CI: 2.35–7.47). Tuscan patients were more likely to escape to non-border regions for HS DRGs while for Musculoskeletal Diseases (in 2019 OR: 0.76; 95%CI: 0.68–0.84; in 2022 OR: 0.83; 95%CI: 0.72–0.89) they were less likely to be admitted to non-border regions for HS DRGs (*p* < 0.05).

**Conclusion:**

The findings highlight the need to implement interregional agreements to optimize patient mobility, avoid duplication of services, reduce costs and improve access to care.

## Introduction

Inter-regional patient mobility is a complex migratory phenomenon of patients using health care services, mainly hospitals, far from their place of residence (1). It can be seen as an opportunity to overcome capacity constraints in the delivery of highly specialized health care services, thereby promoting more efficient, effective and fair care provision. (2). At the European level, patient mobility is primarily governed by Directive 2011/24/EU, which establishes the right of EU citizens to access healthcare services in any member state and to be reimbursed by their home country. This directive facilitates cross-border healthcare, enabling patients to seek medical treatments abroad, particularly when long waiting times or the unavailability of specific treatments in their home country pose significant barriers (3, 4). In the single-payer public and universalistic Italian National Health Service (NHS) where health federalism is in force and where the citizen is free to choose the place of treatment, both public and private providers agreed upon NHS; without any geographic constraints this becomes even more relevant as a financial transaction is created between the regions of residence and destination, respectively (5, 6). For this and the growing increase in this phenomenon, its analysis has lately become an increasingly hot topic for policy makers (1, 7). In this context, there is inevitable competition both between hospitals in the same region and between different regions (8). Already at the European level, competition exists in several countries and is facilitated by activity-based payment systems, often based on DRGs Diagnosis-Related Groups (DRGs), where hospitals are reimbursed based on the volume and complexity of cases treated (9, 10). However, except in the Netherlands, there is no competition on prices, which are regulated under various forms of fixed price regulation (11). Despite the presence of competition among hospitals, price control through DRGs was introduced to incentivize hospitals to compete on quality to increase their revenues (12). However, some economic models indicate that competition can have complex effects on quality, which is also influenced by hospitals’ budget constraints (13). It may also incentivize hospitals to reduce costs through comparison competition, but constraints on profits may dampen these incentives (14). The excessive competition can hinder cooperation among providers, as highlighted in France, where a new policy instrument was introduced to encourage cooperation and integration of public hospitals: each hospital must join a group associated with a teaching hospital, and can share activities, equipment, medical teams and a joint information system (14, 15). In Italy, high-volume hospitals, often in less competitive areas, tended to achieve better results due to experienced surgeons and larger caseloads. Patients chose hospitals based on care quality, reinforcing this relationship (16). For CABG surgery, lower risk-adjusted mortality ratios in more competitive areas were consistent with findings from the English NHS, indicating competition can improve care quality (17). In addition, hospital choice based on perceived quality are influenced by outcomes beyond local markets, with significant short- and long-term interdependence. Dynamics of local and global competition among hospitals have been evidenced, suggesting that health systems could improve by promoting competitive local markets and institutional environments for hospital management (18). Therefore, patient migration can be considered a proxy of healthcare quality in a single-payer system such as the Italian one. Also, the choice of moving to another hospital or region can be influenced by the complexity of the care need; in other words, we hypothesize that patient migration is more frequent for highly specialized care rather than low-complexity needs. Considering this, our study aims to analyze the association between border mobility in the Tuscany region (Italy) and patient migration to and from other regions for high-specialty surgical DRGs.

## Materials and methods

### Study design and data sources

A retrospective study was conducted on data of 2019 and 2022 collected from the Hospital Discharge Cards provided by the Italian Ministry of Health. We considered all acute care ordinary admissions of patients residing in the Tuscany region and hospitalized to other Italian regions (escapes) and those of residents of other regions admitted to Tuscan hospitals (attractions). We studied the surgical Diagnosis-Related Groups (DRGs) of each Major Diagnostic Categories (MDCs) according to high specialty and non-high specialty. MDCs that did not have high-specialty DRGs on the list and those in which high-specialty DRGs did not generate admissions were excluded (Table 1).

**Table 1:** MDCs with both high specialty and non-high specialty DRGs included in the study.

MDC	
Number	Description
1	Diseases and Disorders of the Nervous System
3	Diseases and Disorders of the Ear, Nose, Mouth And Throat
4	Diseases and Disorders of the Respiratory System
5	Diseases and Disorders of the Circulatory System
6	Diseases and Disorders of the Digestive System
7	Diseases and Disorders of the Hepatobiliary System And Pancreas
8	Diseases and Disorders of the Musculoskeletal System And Connective Tissue
10	Diseases and Disorders of the Endocrine, Nutritional And Metabolic System
11	Diseases and Disorders of the Kidney And Urinary Tract
13	Diseases and Disorders of the Female Reproductive System
17	Myeloproliferative DDs (Poorly Differentiated Neoplasms)
18	Infectious and Parasitic DDs (Systemic or unspecified sites)
21	Injuries, Poison And Toxic Effect of Drugs

### Analysis

Data was divided in patients admitted in Tuscan hospital from other regions (attractions) and those residing in Tuscany hospitalized in other regions (escapes). For each year and MDCs included in the study, patient attractions and escapes to bordering (Liguria, Emilia-Romagna, Marche, Umbria and Latium) and non-bordering (Aosta Valley, Piedmont, Lombardy, Autonomous Province of Trento, Autonomous Province of Bolzano, Veneto, Friuli-Venezia Giulia, Abruzzo, Molise, Campania, Basilicata, Apulia, Calabria, Sicily and Sardinia) regions with Tuscany were associated with high specialty and non-high specialty surgical discharge DRGs by Odds Ratio. The analysis was carried out with STATA software SE/14.0 (StataCorp LLC, Texas USA). The significance level was set at 95% (*p* < 0.05).

## Results

Tables 2, 3 show the association between attractions in Tuscan hospitals for high specialty and non-high specialty surgical DRGs and bordering and non-bordering regions for the studied years. In 2019, for surgeries related to Diseases and Disorders of the Digestive System (MDC 6), patients from non-border regions were more likely (approximately 37%) to be attracted to Tuscan hospitals for high-specialty DRGs (OR: 1.37; 95%CI: 1.08–1.74; *p* < 0.05). Conversely, for surgeries concerning Diseases and Disorders of the Musculoskeletal System and Connective Tissue (MDC 8), patients from the same regions were less likely (about 49%) to be attracted for high-specialty DRGs (OR: 0.51; 95%CI: 0.47–0.56; *p* < 0.05). In 2022, the associations found for MDC 6 were confirmed (OR: 1.66; 95%CI: 1.29–2.12; *p* < 0.05) as well as those for MDC 8 (OR: 0.53; 95%CI: 0.48–0.59 *p* < 0.05), with increases up to 66 and 53%, respectively, in the likelihood of patients from non-border regions being attracted for high-specialty DRGs. Additionally, in 2022, patients from non-border regions were significantly more likely to be attracted to Tuscan hospitals for high-specialty DRGs in the following categories: Diseases and Disorders of the Nervous System (MDC 1) with a 91% increase (OR: 1.91; 95%CI: 1.30–2.80; *p* < 0.05), Diseases and Disorders of the Hepatobiliary System and Pancreas (MDC 7) with an 84% increase (OR: 1.84; 95%CI: 1.20–2.82; *p* < 0.05), Infectious and Parasitic Diseases (MDC 18) with a remarkable 781% increase (OR: 8.81; 95%CI: 1.61–48.30; *p* < 0.05), and Injuries, Poisoning and Toxic Effects of Drugs (MDC 21) with a 240% increase (OR: 3.40; 95%CI: 1.29–8.24; *p* < 0.05). Conversely, for Diseases and Disorders of the Endocrine, Nutritional, and Metabolic System (MDC 10), in 2022, patients from non-border regions were less likely (about 51%) to be attracted for high-specialty DRGs (OR: 0.51; 95%CI: 0.27–0.59; *p* < 0.05).

**Table 2:** Association between attractions in Tuscan hospitals for high specialty and non-high specialty of surgical DRGs and bordering and non-bordering regions, year 2019.

MDC	OR	95% CI	*p*
1	1.144171	0.863160–1.516668	0.3486
3	1.478353	0.925634–2.361113	0.0996
4	0.578579	0.307197–1.089701	0.0863
5	0.875995	0.719577–1.066414	0.1868
6	1.374508	1.084563–1.741966	**0.0082**
7	1.302632	0.889566–1.907502	0.1730
8	0.510332	0.466813–0.557909	**<0.0001**
10	1.610037	0.798359–3.246933	0.1792
11	0.905215	0.718270–1.140818	0.3986
13	1.611686	0.923788–2.811828	0.0897
17	1.22336	0.450024–2.799050	0.8044
18	3.466667	0.719558–16.701614	0.0986
21	1.037762	0.433862–2.482239	0.9336

**Table 3:** Association between attractions in Tuscan hospitals for high specialty and non-high specialty of surgical DRGs and bordering and non-bordering regions, year 2022.

MDC	OR	95% CI	*p*
1	1.908716	1.298976–2.804666	**0.0008**
3	1.538204	0.937098–2.524893	0.0861
4	1.461538	0.622247–3.432870	0.3809
5	0.830839	0.674154–0.674154	0.0818
6	1.655793	1.291224–2.123296	**0.0001**
7	1.841361	1.200653–2.823971	**0.0045**
8	0.531924	0.481227–0.587962	**<0.0001**
10	0.514920	0.266273–0.587962	**0.0445**
11	1.016119	0.793387–1.301379	0.8992
13	1.433182	0.976460–2.103527	0.0646
17	0.732659	0.291149–1.843691	0.5071
18	8.813815	1.608467–48.307451	**0.0024**
21	3.397059	1.293139–8.924028	**0.0083**

Tables 4, 5 shows the association between escapes from Tuscany for high specialty and non-high specialty of surgical DRGs and bordering and non-bordering regions, year 2019 and 2022. Regarding the escapes in 2019 for surgeries of Diseases and Disorders of the Nervous System (OR: 3.08; 95%CI: 2.18–4.37; *p* < 0.05), of Diseases and Disorders of the Ear, Nose, Mouth And Throat (OR: 2.41; 95%CI: 1.52–5.03; *p* < 0.05) and of Diseases and Disorders of the Hepatobiliary System And Pancreas (OR: 4.55; 95%CI: 2.71–7.68; *p* < 0.05), Tuscan patients were more likely to hospitalize to non-border regions for high specialty DRGs, approximately three times, almost two and a half times, and about four and a half times more, respectively. While for Diseases and Disorders of the Musculoskeletal System and Connective Tissue (OR: 0.76; 95%CI: 0.68–0.84; *p* < 0.05) they were less likely (about 24%) to be admitted to non-border regions for high specialty DRGs. In 2022, the previous associations were confirmed for MDC 1, MDC 7 and MDC 8. For MDC 1 (OR: 2.26; 95%CI: 1.60–3.22; *p* < 0.05) and MDC 7(OR: 4.19; 95%CI: 2.35–4.47; *p* < 0.05), the probability of escape to non-bordering regions for high-specialty DRGs was reduced by 81 and 39%, respectively, while for MDC 8 Tuscan patients probability of being admitted to non-bordering regions for high specialty DRGs increased by 7% (OR: 0.84; 95%CI: 0.72–0.89; *p* < 0.05). In addition, for Myeloproliferative DDs (MDC 17) an 80% probability of being admitted to hospital of non-border regions for high specialty DRGs was observed (OR: 0.20; 95%CI: 0.06–0.70; *p* < 0.05). Finally, for MDC 3 no significant association was shown in the last year of the study.

**Table 4:** Association between escapes from Tuscany for high specialty and non-high specialty of surgical DRGs and bordering and non-bordering regions, year 2019.

MDC	OR	95% CI	*p*
1	3.083260	2.176043–4.368704	**<0.0001**
3	2.408257	1.152695–5.031429	**0.0158**
4	0.443995	0.179142–1.100421	0.0716
5	1.135578	0.904055–1.426392	0.2741
6	1.149999	0.874087–1.513004	0.3176
7	4.557407	2.705255–7.677637	**<0.0001**
8	0.758533	0.681502–0.844272	**<0.0001**
10	0.971719	0.510403–1.849983	0.9304
11	0.967083	0.659921–1.417215	0.8637
13	0.811793	0.364717–1.806899	0.6089
17	0.791117	0.355047–1.762769	0.5656
18	5.142857	0.686559–38.523959	0.0754
21	2.058824	0.451005–9.398469	0.3408

**Table 5:** Association between escapes from Tuscany for high specialty and non-high specialty of surgical DRGs and bordering and non-bordering regions, year 2022.

MDC	OR	95% CI	*p*
1	2.267852	1.598998–3.216485	**<0.0001**
3	0.929412	0.409450–2.109674	0.8610
4	0.938976	0.590106–1.494098	0.7904
5	1.158983	0.894468–1.501720	0.2639
6	1.094553	0.823759–1.454365	0.5331
7	4.187192	2.347027–7.470122	**<0.0001**
8	0.833268	0.721335–0.894507	**0.0001**
10	1.558158	0.747044–3.249949	0.2332
11	1.268428	0.861613–1.867323	0.2271
13	1.556273	0.992840–2.439453	0.0518
17	0.204290	0.059619–0.700014	**0.0051**
18	1.600000	0.287128–8.915864	0.5884
21	1.688312	0.571563–4.987019	0.3378

## Discussion

Inter-regional s mobility is a complex and steadily growing phenomenon that indirectly indicates a regional health service’s ability to meet its residents’ need for care locally (1, 19). In a single-payer system such as Italy’s, patient flows across regional borders acts serves as an indicator of perceived healthcare quality: when care needs are highly complex, people are more likely to seek treatment elsewhere (20). In addition, several factors can play a role in patient willingness to travel for care: hospital capacity, which offers certainty of admission; the presence of advanced technology, perceived as a guarantee of better care; high organizational efficiency, which reduces waits and costs; and, finally, ease of logistical access to the region itself. Patient flows are constrained by high per capita Gross Domestic Product and by geographic distance, although the latter can be offset if the destination enjoys a strong reputation. Furthermore, positive externalities (spatial lags) influence the phenomenon, as regions tend to emulate their more efficient neighbors, thereby attracting extra-regional patients (21). Nowadays, the value of healthcare mobility is €5,037 million the highest since 2010 with an increase of 18.6% over 2021. In this context, Tuscany is among the seven most “virtuous” regions with positive mobility balance (22). This study analyzes patient mobility in Tuscany for surgical DRGs, examining associations between border/non-border migrations and high or non-high specialty care. Italian regions vary widely in size and shape: mobility understood as leaving regional boundaries is obviously greater in smaller regions, which tend to have less health facility, than in larger ones and in those with a high perimeter-to-area ratio, Tuscany being among the latter. It is therefore important to distinguish between proximity mobility (hospital, albeit in a different region, is closer to a patient), and medium- to long-range mobility (23). Martini et al. (24) explore the factors influencing patient willingness to travel and highlight the importance of quality and accessibility. Their findings underline the necessity for a tailored approach in healthcare mobility strategies to address these variances across regions. The results reveal notable trends and patterns in inter-regional patient mobility for high-specialty DRGs in Tuscany, highlighting shifts in patient attraction and escape dynamics over time, reflecting complex dynamics with significant clinical and policy implications. The attractiveness of Tuscan hospitals for high specialty DRGs, particularly for MDC 6 (digestive system) and since 2022 for other categories as MDC 1 (nervous system), could be closely linked to high quality and availability of advanced technologies. This aligns with the findings of Carnazza et al. (25) who emphasize how the presence of centers of excellence and private accredited hospitals serve as a strong pull factor for patients from other regions, especially those from economically disadvantaged southern areas of Italy. In addition, this mirrors Berta et al.’s insights (26) into the strategic behaviors of hospitals in Lombardy, which demonstrate how superior perceived quality and reduced waiting times effectively attract patients from other regions, enhancing the reputation and financial stability of healthcare institutions. Tuscany, positioning itself as a leader in certain specializations, benefits from interregional flows that reinforce its role as a healthcare hub. However, certain areas, such as the treatment of musculoskeletal disorders (MDC 8), show lower attractiveness for high specialty, possibly due to the availability of local alternatives or reflecting possible competition from hospitals in other regions with established strengths in orthopedic surgery or a lesser perceived need for interregional travel for such interventions. This latter finding aligns with Frischhut and Levaggi (27) who highlight the significant influence of proximity and patient perceptions in determining mobility patterns, especially in regions near borders. These dynamics underline how localized strengths and patient convenience play crucial roles in shaping healthcare mobility. In addition, this pattern, as noted by Servadio et al. (28), may also stem from inequalities in healthcare access, which discourage less affluent patients or those with fewer resources from traveling. Conversely, the rates of escape to non-bordering regions specifically for MDC 1 and MDC 7 (Hepatobiliary System and Pancreas) reveal another aspect of healthcare mobility: competition among regions to attract high-value patients both economically and clinically. Berta et al. (29) highlight how perceived quality, and social networks influence healthcare mobility, but also how financial incentives and shorter waiting times in other regions can lead patients to choose facilities outside Tuscany. Sicily’s experience with the hub-and-spoke model for neurorehabilitation, as reported by Ielo et al. (30), provides an interesting perspective on how service reorganization can reduce healthcare mobility and improve local access to treatments. Implementing a similar model in Tuscany could help reduce escape rates, especially in rural or underserved areas, and ensure an equitable distribution of advanced healthcare services. The economic implications of these flows are significant. As highlighted by Carnazza et al. (25), healthcare mobility generates interregional transfers that, if not managed adequately, risk widening existing disparities. Tuscany, as a net attracting region, benefits from these flows, but the risk is that less advantaged regions suffer a loss of resources that could further compromise the quality of their services. Finally, the described dynamics highlight the challenges related to the decentralization of the Italian healthcare system. As noted by Maino and Maioni (31), the regionalized Italian system, while ensuring freedom of choice, accentuates territorial and socioeconomic inequalities. In this context, it is crucial to implement compensation and redistribution policies to ensure equitable access to advanced healthcare services across all regions.

### Strengths and limitations

Our study remains highly valuable to public-health decision-makers. By analyzing patients’ willingness to seek care in neighboring and non-neighboring regions, we provide actionable insights for resource allocation and service planning, identifying where—and for which surgical specialties—people are most likely to cross regional borders. Despite the significant findings of this study, it is essential to recognize its limitations. Firstly, the latest aggregated and, therefore, reliable post pandemic data available are up to 2022, so associations could be different in later years. Secondly, the study only analyzes surgical and not medical DRGs, so there might be different results for the latter. In addition, our database did not allow us to stratify by demographic characteristics, so there might be different associations across categories. Finally, the analysis focuses on a single Italian region, and different associations might be observed in other regions.

## Conclusion

This study aimed to analyze the association between border mobility in the Tuscany region (Italy) and patient migration to and from other regions for highly specialized surgical DRGs. From 2019 to 2022, the number of MDC categories with significant associations for attractions increased, while the trend for escapes remained roughly stable. This highlights the evolving dynamics of patient mobility in Tuscany, with an increasing ability to attract patients from non-neighboring regions for highly specialized care, while escape patterns have remained fairly consistent. The dynamics showed in our results underscore the importance of nuanced strategies that balance the attraction of high-quality centralized care with the benefits of localized healthcare solutions. This reinforces the need to implement interregional cooperative agreements to optimize patient mobility, avoid duplication of services, reduce costs, and improve access to care. For such cooperation to be equitably beneficial, it is necessary to maintain a balance between the supply of specialized services and their demand, as well as to ensure the consistent quality of care provided. By adopting well-designed mobility agreements, regions can enhance collaboration, mitigate disparities in healthcare access, and improve the quality of services across Italy. This approach supports the efficient allocation of resources while fostering a more integrated and equitable healthcare system for all citizens. Future research could expand upon this work by examining broader patterns of patient mobility, exploring additional dimensions of this phenomenon, and assessing the long-term impact of mobility agreements on health outcomes and regional healthcare systems.

## Data Availability

The raw data supporting the conclusions of this article will be made available by the authors, without undue reservation.
